# No variation and low synonymous substitution rates in coral mtDNA despite high nuclear variation

**DOI:** 10.1186/1471-2148-6-24

**Published:** 2006-03-16

**Authors:** Michael E Hellberg

**Affiliations:** 1 Department of Biological Sciences, Louisiana State University, Baton Rouge, LA 70803, USA

## Abstract

**Background:**

The mitochondrial DNA (mtDNA) of most animals evolves more rapidly than nuclear DNA, and often shows higher levels of intraspecific polymorphism and population subdivision. The mtDNA of anthozoans (corals, sea fans, and their kin), by contrast, appears to evolve slowly. Slow mtDNA evolution has been reported for several anthozoans, however this slow pace has been difficult to put in phylogenetic context without parallel surveys of nuclear variation or calibrated rates of synonymous substitution that could permit quantitative rate comparisons across taxa. Here, I survey variation in the coding region of a mitochondrial gene from a coral species (*Balanophyllia elegans*) known to possess high levels of nuclear gene variation, and estimate synonymous rates of mtDNA substitution by comparison to another coral (*Tubastrea coccinea*).

**Results:**

The mtDNA surveyed (630 bp of cytochrome oxidase subunit I) was invariant among individuals sampled from 18 populations spanning 3000 km of the range of *B. elegans*, despite high levels of variation and population subdivision for allozymes over these same populations. The synonymous substitution rate between *B. elegans *and *T. coccinea *(0.05%/site/10^6 ^years) is similar to that in most plants, but 50–100 times lower than rates typical for most animals. In addition, while substitutions to mtDNA in most animals exhibit a strong bias toward transitions, mtDNA from these corals does not.

**Conclusion:**

Slow rates of mitochondrial nucleotide substitution result in low levels of intraspecific mtDNA variation in corals, even when nuclear loci vary. Slow mtDNA evolution appears to be the basal condition among eukaryotes. mtDNA substitution rates switch from slow to fast abruptly and unidirectionally. This switch may stem from the loss of just one or a few mitochondrion-specific DNA repair or replication genes.

## Background

Rates of nucleotide substitution for mitochondrial DNA (mtDNA) are several times higher than those for nuclear DNA (nDNA) for most animals. Several reasons for this difference in rates have been proposed. Because mtDNA is haploid and usually maternally inherited, the effective population size of mitochondrial genes is one quarter that for nuclear genes, which should speed neutral divergence [1]. Unlike nDNA, mitochondria lack histones, which leaves them exposed to mutagens. Furthermore, because mitochondria are centers of oxidative metabolism, mtDNA faces more of the free radicals responsible for many mutations than does nDNA [2]. As a result, intraspecific variation for mtDNA may exceed that for nDNA [3]. This variation is often partitioned among populations, such that surveys of mtDNA may reveal population subdivision when other markers such as allozymes do not [4-7].

Yet not all mtDNA evolves rapidly. Rates of nucleotide substitution are slow in the mtDNA of plants, both in absolute terms and relative to nDNA [8]. Rates of mtDNA substitution appear to be slow in some basal animals as well, including sponges [9,10] and anthozoans (corals, anemones, and their kin, [11-15]). This is especially surprising for corals. Corals and other anthozoans do not sequester their germ cells [16], yet single coral colonies may live many hundreds of years [17]. All else being equal, this combination of a nonsequestered germline and great longevity should lead to high rates of mitochondrial mutation, as any mutations accumulated over a long life could be passed on to offspring.

Contrary to this expectation, mtDNA divergence among closely related anthozoans is low [14,18,19], in fact lower than that for nDNA from the same taxa [20,21]. Among the few intraspecific studies to survey mitochondrial variation from many (>20) individuals, most have focused on non-coding regions [22] or rRNA genes [23]. Variation in these regions has generally been less than or equal to that for nuclear genes [24,25].

Such non-coding substitution rates are difficult to compare across taxa due to the effects of variation in evolutionary constraints on substitution rates and to difficulties in aligning homologous sites. Synonymous (silent) substitutions within coding regions, however, can often be aligned unambiguously among distant taxa. Synonymous sites show low variation in rates among different loci in the same genome [26], consistent with their neutral evolution. To date, surveys of mitochondrial coding regions in corals [25,27,28] have found little or no variation, but these studies have included just a few individuals (≤ 8) and a few localities (≤ 3), and have not been accompanied by surveys of variation at single-copy nuclear markers.

Here, I survey intraspecific variation and estimate the interspecific divergence rate for two species of coral, *Balanophyllia elegans *and *Tubastrea coccinea*, using a widely sequenced mitochondrial coding region from cytochrome *c *oxidase subunit I (*coxI*). Allozyme surveys in *B. elegans *have previously established that single copy nuclear markers are both variable and subdivided among populations [29,30]. Divergence between the two species surveyed allows me to calculate fossil-calibrated rates of synonymous substitution and to place these rates of mtDNA substitution in the phylogenetic context of synonymous rates in other eukaryotic lineages

## Results

*coxI *was invariant among all 67 *B. elegans *(GenBank Accession DQ445805) sampled from 18 populations spanning 3000 km of its geographic range, including 16 individuals from the site (Bodega Bay) that was most polymorphic for allozymes [29]. The seven *T. coccinea *samples from the Caribbean and the Eastern Pacific were likewise identical at *coxI *(Accession DQ445806). Rate calculations were based on this Caribbean/Eastern Pacific consensus sequence. The five sequences from Hawaiian *T. coccinea *were identical to each other, but differed from the Caribbean/Eastern Pacific consensus sequence by a single nonsynonymous substitution. Reading frames for all sequences remain open for all of these sequences when translated using the cnidarianmitochondrial genetic code [31].

The fossil record indicates that *Balanophyllia *and *Tubastrea *diverged at least 50 MY ago [32], yet raw *coxI *divergence between *B. elegans *and *T. coccinea *is only 2.7%. The synonymous substitution rate is 0.00055 substitutions per site per MY (Table 1). This rate is the same as that for two angiosperms (rice and maize) that diverged at about the same time as *Balanophyllia *and *Tubastrea *[26]. For comparison, diverse animal lineages sundered by the Isthmus of Panama three MY ago show rates of synonymous substitution roughly 100 times greater than for angiosperms and corals (Table 1); even the notably slow mtDNA of sharks [33] is 50 times faster. Nonsynonymous (amino acid altering) substitution rates for corals and plants are similar as well, but are only slightly slower than those bilateral animals (Table 1).

**Table 1:** Rates of nucleotide substitution in protein-coding mtDNA from corals, plants, and bilateral animals.

	Time of divergence (MY)	K_S_/yr^a^	K_A_/yr^a^
*B. elegans *vs. *T. coccinea *(corals, Anthozoa)	50	0.056 (0.020)	0.019 (0.006)
Rice vs. maize[26] (Angiosperms)	50	0.05 (0.01)	0.02 (0.00)
*Tegula verrucosa *vs. *T. viridula*[36,79] (topsnails; Lophotrochozoa)	3	5.7 (0.93)	0.033 (0.033)
*Alpheus panamensis *vs. *A. formosus*A[80] (snapping shrimp; Ecdysozoa)	3	8.6 (1.38)	0.0
*Echinometra vanbrunti *vs. *E. lucunter*[81] (sea urchins; invertebrate Deuterostoma)	3	8.1 (1.27)	0.036 (0.036)
*Abudefduf saxatilis *vs. *A. troschelli*[82] (damselfish; vertebrate Deuterostoma)	3	4.5 3 (0.36)	-
*Sphyrna tiburo tiburo *vs. *S. t. vespertina*[33] (sharks; vertebrate Deuterostoma)	3	2.4 (0.41)	0.056 (0.033)

^a ^Rates are in units of % substitutions per site per 10^6 ^years. K_S_, synonymous substitutions per synonymous site; K_A_, nonsynonymous substitutions per nonsynonymous site. All estimates for fragments of *coxI *except angiosperms (mean of several mitochondrial genes, including *coxI*) and *Sphyrna *(*cytochrome b*).

Patterns of nucleotide substitution between the two corals are also more similar to angiosperms [8] than to other animals. About half of coral mtDNA substitutions are transversions (9/17). In contrast, the mtDNA of other animals typically shows a strong transition bias [34,35]. For example, the *Tegula *species in Table 1 show a 15-fold excess of transitions compared to transversions between closely-related species [36].

## Discussion

### Low levels of mitochondrial variation in corals

This study revealed little intraspecific variation within either of two corals, *Balanophyllia elegans *or *Tubastrea coccinea*. *B. elegans *was sampled over much (> 3000 km) of its broad geographic range, where high levels of allozyme variation have been found previously [29,30]. The single substitution (a nonsynonymous one) observed within *T. coccinea *mtDNA occurred between populations separated by over 4000 km of uninhabitable ocean. Previous surveys of genetic variation in coding regions of coral mtDNA have found similar patterns: either no variation [27] or very little variation restricted to nonsynonymous sites [28].

Such low levels of genetic variation are not characteristic of anthozoan nuclear genes. Indeed, a comparison of allozyme polymorphism and heterozygosity found variation in cnidarians and sponges to be higher than those for all other animals [37]. High allozyme heterozygosity in *B. elegans *[29,30] shows it is no exception to this trend. Intraspecific surveys of nDNA sequence variation from coding regions are lacking for corals, but intron sequences are quite variable [20,24,38], and microsatellites [39] and AFLPs [25] have revealed both high heterozygosities and population subdivision. While more extensive surveys of nucleotide variation from coding nDNA are needed, low variation appears to be restricted to the mitochondrial genome of corals.

Relatively low levels of mtDNA variation can result from range expansions, where the smaller effective population size of mtDNA genes can enhances founder effects. This may account for the lack of variation between Eastern Pacific and Caribbean *T. coccinea*, if the latter were indeed recently introduced as suggested by Cairns [40]. This does not appear to be the case for *B. elegans*, however. Subdivision within this species [29] does not suggest any anthropogenic range changes. Natural poleward range expansions following climatic cooling events can homogenize the mtDNA of newly founded populations, but more equatorial populations continue to harbor variation [41]. Sampled populations of *B. elegans *include its southern range limit, however, but still reveal no variation. Selective sweeps can also homogenize mtDNA within species. However, mtDNA regions are often identical among different species, genera, and even families [21,42], a pattern that would require very strong stabilizing selection to maintain homogeneity (even at silent sites) over millions of years. The most likely explanation of low levels of mtDNA variation within coral species, then, is a low rate of nucleotide substitution.

### Slow rates of synonymous substitution in corals

A growing body of evidence suggests that the mtDNA of anthozoans evolves slowly [11-15,19]. The very low divergence found here for two genera with independent fossil records extending back over 50 MY provides an estimate of just how slowly: 0.055% per MY. This rate of synonymous substitution is 50–100 times slower than those reported previously for an array of animals (Table 1), including hydrozoans [43].

**Figure 1:**
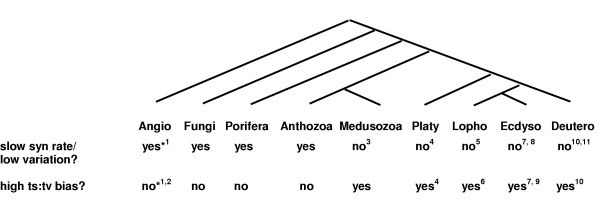
Phylogenetic correlation of tempo and mode of nucleotide substitution in mitochondrial DNA for flowering plants (Angio = Angiosperms), Fungi, Porifera (sponges), Cnidarians (Anthozoans and Scyphozoans), and bilateral animals [Platy = Platyzoa (flatworms), Lopho = Lophotrochozoa (molluscs, annelid worms, bryozoans), Ecdysozoa (arthropods, nematodes), and Deutero = Deuterostoma (echinoderms, tunicates, vertebrates)]. Supporting data: 1 [49] *see text for exceptions; 2 [8]; 3 [43,56]; 4 [59]; 5 [79,83]; 6 [36]; 7 [35]; 8 [80]; 9 [84]; 10 [34]; 11 [81,85].

These low rates have practical consequences. First, the dearth of variation in anthozoan mtDNA makes routine phylogeographic surveys impossible. Alternative approaches employing microsatellite variation have revealed genetically isolated regions within coral species [39], but primers for single-copy nuclear gene regions that both amplify across diverse taxa and consistently reveal variation within species remain to be developed. Second, low variation means that mtDNA sequences cannot be counted on to reveal differences between closely related species. Indeed, the mtDNA region nominated for such DNA barcoding [44] is the very same *coxI *used here. Hebert et al. [45] stated previously that barcoding fails in cnidarians due to low variation; Figure 1 suggests that this limitation applies only to a subset of the Cnidaria, the anthozoans. Still, low rates of *coxI *evolution in corals, sea fans, and sponges mean that the very bricks and mortar of tropical reefs are not amenable to barcoding diversity surveys (although *cox1 *should still prove useful for resolving deeper phylogenetic relationships, e.g. [21]).

### Multiple losses of mtDNA repair function

As with corals, the available data for fungi [46] and sponges [47] suggest that rates of synonymous substitution in mtDNA are slower than nDNA in these taxa. Taken together with rates for plants and animals, these data suggest that mtDNA evolves in two distinct modes: one slow relative to nDNA and with little bias toward transitions, the other fast relative to nDNA and often (but not always, [48]) transition-biased (Figure 1). Phylogenetic analysis suggests that the switch from the slow mode to the fast mode occurs abruptly (without any apparent intermediate state) and always in the direction toward the fast mode. This switch has occurred at least four times: twice in flowering plants (in geraniums and plantains, [49]), and twice in animals (Figure 1).

One change in animals occurred within the phylum Cnidaria between the basal Anthozoa [50,51] and the derived Medusozoa (hydroids and jellyfish). The independently derived fast mtDNA rates in the Medusozoa are similar to those in bilateral animals. In fact, 16S divergence rates in hydroids of the genus *Hydractinia *[52] are greater that of their hermit crab hosts [53], with whom they have probably co-speciated [54]). Phylogenetic analyses reveal interspecific variation typical for most animals among recent radiations of scyphozoans [55]. Scyphozoans also have high levels of intraspecific variation [56,57] and show a transition:transversion bias > 10:1 (e. g. *Cassiopea coxI *data from [58]). A second switch from slow to fast mtDNA occurred at the base of the Bilateria (which includes the vertebrates); even flatworms show high intraspecific mtDNA variation and a strong transition bias [59].

This qualitative switch in the mode of mtDNA evolution is not a simple extension of the quantitative variation in mtDNA rates noted previously [33]. Within vertebrates, relative substitution rates in mtDNA and nDNA are correlated within taxa, despite variation in absolute rates among taxa [60]. Even in bilateral animals with notably slow mutation rates (e.g. the ancient asexual ostracod *Darwinula stevensoni*, [61]), the rate of mitochondrial substitution remains faster than that for nuclear loci. In contrast, the relative rates of substitution in mtDNA and nDNA are reversed in angiosperms, fungi, anthozoans, and probably sponges (nDNA evolves an order of magnitude *faster *than mtDNA) when compared to bilateral animals (where nDNA evolves an order of magnitude *slower *than mtDNA, [34,35]).

No attribute of the mitochondrial genome itself correlates obviously with slow, unbiased evolution. For example, anthozoan mtDNA has group I introns [31,62] like some plants and fungi, but is compact in size (ca. 16–18 kb) like most other animals [63]. Instead, the abrupt and unidirectional switch from slow to fast modes of mtDNA substitution is consistent with the sudden loss of one or a few mitochondrion-specific DNA repair or replication genes. A possible candidate gene has been proposed previously to account for low variation in anthozoans [11,12]: an ortholog of the mismatch repair gene MSH1 (but see [64]). MSH1 is known to be mitochondrion-specific in yeast [65], and is present in the mitochondrial genome of octocorallian anthozoans [66] but missing from the bilateral animals whose genomes have been sequenced. Loss of any of the many genes involved in repair [67] could potentially speed synonymous substitution rates. Note also that the mitochondrial location of the putative MSH1 homolog in octocorallians is exceptional; MSH1 is not present in the mitochondrial genome of corals [19,62], and mitochondrion-specific repair genes are generally encoded in the nuclear genome.

Regardless of the particular genes responsible, a loss of mtDNA-specific repair function (or crippling of mtDNA replication genes) could explain observed low levels of mtDNA variation and divergence compared to nuclear genes. Differences in the fidelity of mtDNA repair and replication could have broad implications. If mutations to mtDNA caused by oxidative stress promote cellular aging [68] and organismal senescence [69-71], then the loss of mtDNA repair abilities may place a physiological ceiling on longevity. Exploring this possibility will require phylogenetic comparisons of covariation in synonymous rates of substitution in mtDNA and patterns of senescence, as well as closer examination of the molecular mechanisms of mtDNA replication and repair across slow and fast mitochondrial lineages.

## Conclusion

Both of the corals surveyed here showed low variation in mitochondrial coding gene sequence, despite the demonstration of extensive nuclear gene variation at allozymes in one of them previously. Combined with other types of nuclear variation (AFLPs, ITS, microsatellites) and phylogenetic studies reported previously, these results suggest that the lack of variation in coral mtDNA results from mechanisms specific to the mitochondrial genome. Synonymous substitution rates suggest coral mtDNA evolves at rates typical for plants, but about 100 times slower than for most animals.

## Methods

### *Balanophyllia elegans *and *Tubastrea coccinea*

*Balanophyllia elegans *is a small solitary coral common at shallow depths in temperate waters ranging between northern Baja California and southeastern Alaska. Average longevity for *B. elegans *has been estimated at about 8.5 years [72]. Dislodgment and overgrowth by algae appear to be the principle causes of death in *B. elegans*; adults do not appear to senesce nor do the growth rates of large individuals slow [72].

The larvae of *B. elegans *crawl along the seafloor during their brief dispersal [73]. As expected given such limited larval dispersal, genetic subdivision (inferred using allozymes) is high between localities separated by hundreds or thousands of kilometers (*F*_ST _= 0.28, [29]). The allozyme markers employed in these genetic surveys were highly variable, both in terms of their mean heterozygosity (0.3, ranging above 0.5 for some loci at some locations) and number of alleles per locus (2.5). These markers have been demonstrated as single copy and Mendelian using controlled crosses [74].

Like *B. elegans*, *Tubastrea coccinea *belongs to the family Dendrophylliidae and broods its larvae. *T. coccinea *is currently recognized as a single species with a circum-tropical distribution [32], however this species has a long history of taxonomic splitting and synonymization. The Atlantic form is morphologically indistinguishable from the Pacific *T. coccinea *and does not appear in the Caribbean fossil record, suggesting this species may have been recently reduced to the Atlantic [40,75]. However these populations appear to be differentiated at the allozyme level (E. Weil, pers. comm.); this Atlantic form has been called *T. aurea*.

### Population sampling

Samples of *B. elegans *were collected from 18 localities spanning over 3000 km of the Pacific coast of North America, namely: Moresby Island, McInnes Island, Stubb's Island, Nanaimo and Bamfield from British Columbia; Tatoosh Island from Washington; Cape Arago from Oregon; Trinidad Harbor, Caspar, Bodega Bay, Santa Cruz, Monterey, San Simeon, Goleta, East Anacapa Island, and Point Loma from California; and Punta Banda and Isla San Geronimo from Baja California.(see [29]). Three individuals were selected for sequencing from each locality, each from a different subpopulation within that locality. In addition, two individuals were sequenced from each of eight subpopulations (a total of 16) from Bodega Bay, where the highest level of allozyme variation occurred (*H *= 0.42, averaged over seven loci [29]).

*T. coccinea *were collected from four localities: Kaneohe Bay, Oahu, Hawaii (5 individuals); La Paz and Isla Cerralvo in Baja California Sur, Mexico (2); Margarita Reef, Isla Magueyes, Puerto Rico (1); and four localities along the coast of Curaçao (4). All samples were taken from 5–15 m depth.

### PCR amplification, sequencing and analysis

DNA was extracted from coral samples using the QIAamp DNA Mini Kit (Qiagen). A 710-bp fragment of *coxI *was initially amplified from *B. elegans *using primers LCO1490 and HCO2198 of Folmer et al. [76]. The resulting sequence was used to design an internal primer (Lc2COI, 5'-CGTTATTTTAGTATTTGGGATTGG-3') that was used in combination with HCO2198 for all subsequent amplification and sequencing.

Amplification products were sequenced directly on an ABI 377 using Big Dye Terminator chemistry, except for six templates (one from an Anacapa *B. elegans*, and *T. coccinea *from Puerto Rico and Curaçao plus three from Hawaii), which were cloned before sequencing. Multiple sequences were obtained from these clones to avoid misinterpretation of PCR errors.

### Calculation of substitution rates

Genetic distances and transition/transversion ratios were estimated using MEGA3 [77]. Jukes-Cantor estimates were used because all mitochondrial sequences were similar (< 3 % divergence from raw counts) and no strong transition bias was evident. Numbers of synonymous and nonsynonymous sites were estimated using the methods of Nei and Gojobori [78], making appropriate adjustments for taxon-specific variation in the mitochondrial genetic code. Rates for trans-Ismuthian pairs were calculated using GenBank sequences for *Tegula *([36]: AF080668, [79]: AF132340), *Alpheus *([80]: AF309923, AF309904), *Echinometra *([81]: AF255539, AF255502) and *Sphyrna (*[33]: L08042, L08043). Rates for the trans-Ismuthian teleost pair *Abudefduf saxatlis *and *A. torschelli *were taken from the literature [82]; sequences for *A. saxatlis *have not been deposited in GenBank and so nonsynonymous rates could not be calculated for this pair. Transition/transversion ratios for scyphozoans were calculated using *Cassiopea *sequences from [58](AY319448-AY319473).

## Abbreviations

mitochondrial DNA (mtDNA); nuclear DNA (nDNA); cytochrome oxidase subunit I (*coxI*); MY (million years)
